# Investigation of the structure of anti-human seminal plasma protein single-chain antibody and its association with linker peptide length

**DOI:** 10.3892/mmr.2015.3980

**Published:** 2015-06-22

**Authors:** XIN JIANG, JUN ZHAI, DONGKUI SONG, QINGSHAN QU, MING LI, LI XING, SHUZHAI MIAO

**Affiliations:** 1 Department of Urology, First Affiliated Hospital of Zhengzhou University, Zhengzhou, Henan 450052, P.R. China; 2 Department of Kidney Transplantation, People's Hospital of Zhengzhou, Zhengzhou, Henan 450003, P.R. China; 3 Reproductive Center, First Affiliated Hospital of Zhengzhou University, Zhengzhou, Henan 450052, P.R. China

**Keywords:** seminoprotein, single-chain antibody, linker, diffusion radius, fore and aft distance

## Abstract

To enhance the activity of seminoprotein single-chain variable fragment (γ-Sm-ScFv) antibodies, modulation of the length of the linker peptide, which connects the variable region of the heavy chain (VH) and the light chain (VL) of single-chain antibodies, was performed in the present study. Homologous modeling of single VH and VL were performed, respectively. Subsequently, modeling of the whole ScFv sequence, which was previously modified with added linkers of different lengths was also performed, and the (Gly_4_Ser)_n_ peptide chain structure was used as the linker. The similarities between VH and VL prior to and following the addition of the linker were compared by applying the algorithm of protein similarity, based on spherical coordinates layering. In addition, changes in the fore and aft distance, and diffusion radius were calculated using a MATLAB tool, based on which changes in structural stability were analyzed. Finally, the single-chain antibody was assessed in a nude mouse model. When n=3 or n=6, the similarity between the original distance and VH and VL were the highest, and the fore and aft distance and diffusion radius were relatively close. In addition, the nude mouse model indicated that, when n=3 or n=6, the inhibitory rate of the single-chain antibody against tumor cells was significantly higher, compared with the other linker peptides of different lengths. The effect of structural changes of the linker peptides in the single-chain antibodies on the whole antibody molecule was examined at different levels using a combination of mathematical modeling, bioinformatics methods and biological experiments. The findings of the present study may provide a foundation for further investigation into the preparation of single-chain antibodies.

## Introduction

**Figure 1:**
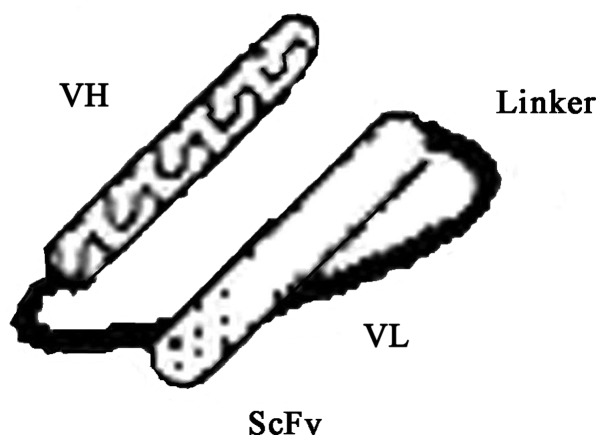
Structure of the ScFv antibody. VH indicates the heavy chain and VL indicates the light chain. ScFv, single chain variable fragment.

Prostate cancer refers to epitheliogenic malignant tumors of the prostate. What is usually termed prostate cancer refers to adenocarcinoma of the prostate. Since the mid to late 1980s, the morbidity and mortality rates of prostate cancer in certain developed areas in China have continued to increase (1). Previous studies have indicated that seminal plasma protein is one of the specific markers of prostate cancer (2). Hao and Liang (3) used 131I to mark the anti-human seminal plasma protein single-chain antibody (γ-Sm-McAb), which was used for the radioimmunoimaging and treatment of prostate cancer. This method was confirmed to be of high sensitivity and specificity, and of beneficial therapeutic effect (3). However, the monoclonal antibodies used originated from murine animals, the repeated use of which may generate anti-mouse antibodies, affecting the therapeutic effect and resulting in allergic reactions (4). Due to the small molecular size, low antigenicity and advantages over other parental antibodies when used within the body, a single chain variable fragment (ScFv) can be used to effectively avoid deficiencies in monoclonal antibodies and has received increasing attention (5). A ScFv is composed of a heavy chain (VH) and a light chain (VL) through linkers of several amino acids (6) (Fig. 1). VH and VL are the minimum functional fragments in the binding site of antibodies to antigens, while the linker peptide is used predominantly for connection between the two for fusion expression. (Gly4Ser)n is usually selected as the linker peptide in ScFv (7), however, the effect of the of the selected n-value on the functional expression of the two variable regions in ScFv is key to investigating the optimal construct of ScFv. In the present study, a similarity algorithm of spherical coordinates for layered proteins was used. Comparison of changes in the structural models of the changeable structures in single-chain antibodies under different values of n, enable the optimal linker length to be determined for maintaining active seminal plasma protein single-antibodies. The results of the present study aim to provide a foundation for the preparation of a single-chain antibody, which may be effective in the treatment of prostate cancer.

## Materials and methods

### Preparation of monoclonal antibody

The anti-human seminal plasma protein hybridoma cell strain was provided by The First Affiliated Hospital of Zhengzhou University (Zhengzhou, China), which was used for secretion of the monoclonal antibody of anti-human seminal plasma protein. Plasmids of *Escherichia coli* JM109 (pUC19) were preserved in the laboratory (First Affiliated Hospital of Zhengzhou University). The restriction endonuclease, Taq DNA polymerase, dNTP, buffer, purification system of Wizard™Plus Minipreps and the expression vector of the Glutathione S-transferase fusion protein (pGEX-4T-1) were purchased from Promega Corporation (Fitchburg, WI, USA); The rapid DNA ligation kit was purchased from Boehringer Mannheim GmbH (Basel, Switzerland); The Advantage™PCR-Pure kit was purchased from Clontech Co. (Tokyo, Japan).

The key instruments used in the present study included a polymerase chain reaction (PCR) DNA Amplifier, a pipettor and refrigerated centrifuge (Eppendorf, Hamburg, Germany), a GFL-7601 incubator (GFL Company, Lower Saxony, Germany), a water bath (Shanghai Senxin Biological Technology Co., Ltd., Shanghai, China), a horizontal electrophoresis apparatus, a gel imaging system (Liyyi Company, Beijing, China) and a −80°C ultra cold storage freezer (Haier, Beijing, China).

### Extraction and reverse transcription of total cellular (c)DNA

The extraction of total cDNA was performed was performed using the DNA one step extraction method, as described previously (8). The total RNA was extracted from the cells and dissolved in 30 *µ*l RNase-free water, and reverse transcription was performed using 5 *µ*l, according to the manufacturer's instructions (Promega Corporation).

### Amplification of variable region genes of the monoclonal antibody

The genes of VH and VL were amplified using 672 ng/*µ*l template cDNA. The primer sequences were as follows: VH, reverse 5′-AGGT (CG) (AC) A (AG) CTGCAG (CG) AGTC (AT) GG-3′ (degenerate primer) and forward 5′-TGAGGAAACGGTGACCGTGGTCCCTTGGCCCCAG-3′; VL, reverse 5′-GTGAATTCGACATCGTGATGACCCAGTCTCC-3′ and forward 5′-CAGTCGACTAACGTTTGATCTCCAGCTTGGTCCC-3′ (Sangon Biotech, Shanghai, China). The PCR reaction system (20 *µ*l) included cDNA (1 *µ*l), dNTP (0.4 *µ*l), upstream and downstream primers (each 0.3 *µ*l), Taq DNA polymerase (0.2 *µ*l), buffer (2 *µ*l) and sterilized water (15.8 *µ*l). The reaction conditions included a denaturing stage for 60 sec at 94°C, annealing for 90 sec at 55°C, extension for 120 sec at 72°C, for 30 cycles. Following extracting 5 *µ*l amplicon from each of the VH and VL samples to perform the lipid sugar electrophoresis detection, the remaining amplicon was recycled by Advantage™PCR-Pure kit according to the manufacturer's instructions and then was sequenced by the Shenggong Bioengineering Limited Company (Shanghai, China).

### Modeling

The corresponding amino acid sequence was acquired by translating VH and VL gene sequences in γ-Sm-ScFv. The amino acid sequence of the protein determined the advanced structure. Firstly, tertiary-structure modeling of VH and VL were performed using the homologous modeling method. The amino acid sequences of VH and VL were sent to SWISS-MODEL for modeling (http://www.swissmodel.expasy.org/), and the acquired data file of the protein data bank structure was treated as the original structural file.

A total ScFv sequence can be combined by adding linker amino acid sequences and connecting them between VH and VL. Among which, (Gly4Ser)n was selected for the linker sequence. (Gly4Ser) refers to five short amino acid peptides, which are composed of four glycines and one serine. This oligopeptide is a repeated unit in the linker peptide, which is used for ligation of the VL and VH segments. It has relatively high flexibility or bending capacity. The small steric hindrance is useful for the interaction of the VL and VH segments, and arrangement of the correct conformation, as well as improving the stability of antibodies. Such structure is not easily recognized or degraded by proteases, which contributes to the stability of antibodies within the body. The combinatorial ScFv-n sequence was also used in the modeling, and the data of the overall three-dimensional structure and that of VHn and VLn were obtained.

### Structural contrast

To investigate the effect of the length of the linker peptide on the structure of VH and VL, the structural similarity of VH and VL in (Gly4Ser)n was calculated at different values of n. In addition, a space spherical shell hierarchical matching algorithm, based on spherical coordinates and described by Zhang and Chen (9), was used to calculate the level of similarity. In this algorithm, the protein was treated as the spherome, and the euclidean coordinates of each atom in the protein were transformed into spherical coordinates. According to the radius, the protein was then divided into several layers of spherical shells, and the same number of same atoms in each layer were collected. On the basis of predetermined weights, the number of atoms were determined, thus, obtaining the final value of atoms in each layer. Each layer of atomic values of VH-(Gly4Ser)n or VL-(Gly4Ser)n and VH (or VL) were stacked into vector a and vector b, respectively. The included angle cosine function was treated as a similarity function. The formula used (1), the algorithm of which was achieved using MATLAB 2012a software (The MathWorks, Inc., Natick, MA, USA) was as follows:

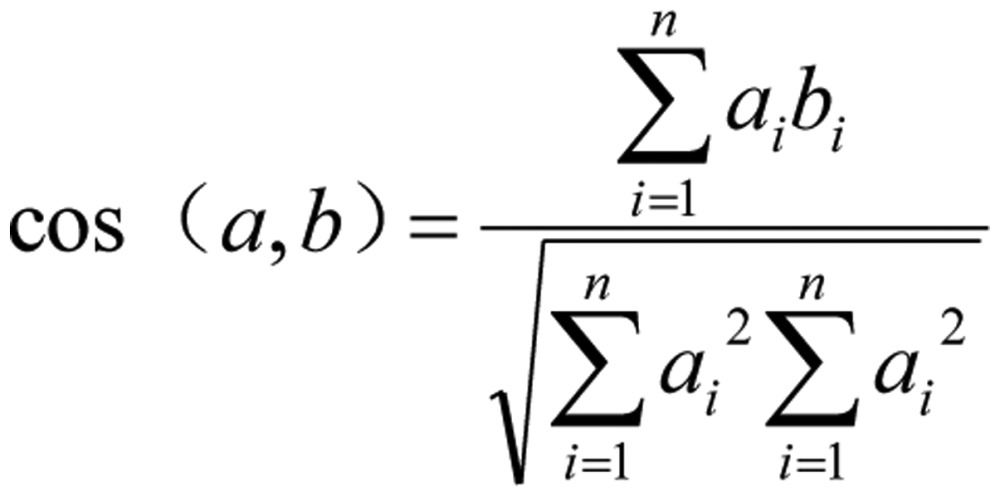


### Stability analysis

The stability of ScFv is presented as the fore and aft distance or the diffusion radius. The fore and aft distance refers to the distance from the fore α-carbon atom to the aft α-carbon atom. Using MATLAB, the fore and aft distances of the original structure of VH and VL were calculated, respectively, adding the fore and aft distances of VHn and VLn when n=0–9. In addition, their largest diffusion radius was calculated.

## Results

**Figure 2:**
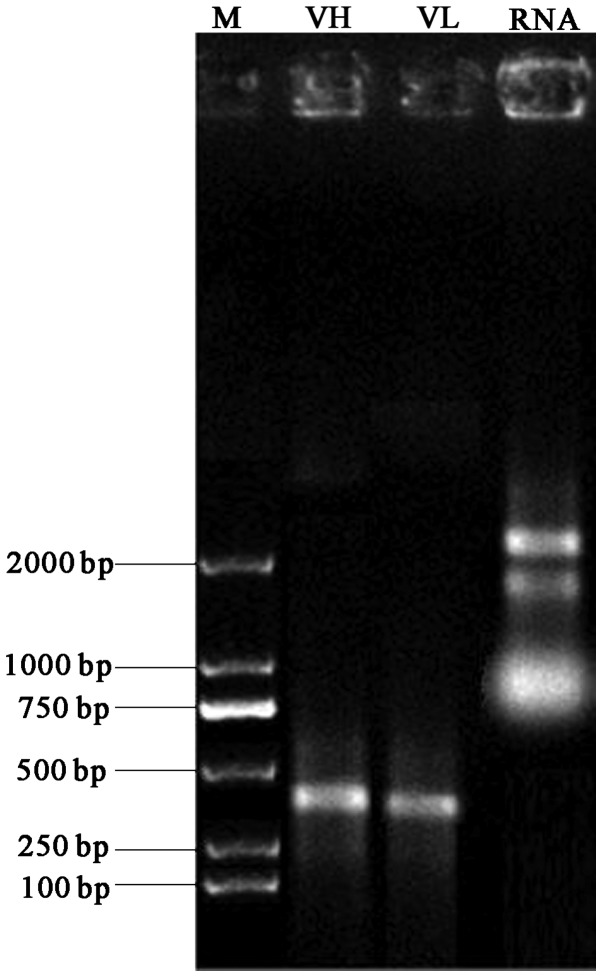
Gel electrophoresis, performed with separate separation of VH and VL. VH, heavy chain; VL, light chain; M, marker.

The optical density (OD)_260_/OD_280_ value of the overall extracted mRNA was 2.1, according and its concentration was 1,279 ng/*µ*l, which met the requirements of the experiment. The concentration of cDNA transcribed by reverse transcription was 672 ng/*µ*l. The cDNA was diluted at 1:50 and was used to amplify of the VH and the VL of the seminal plasma protein's single-chain antibody using PCR. The results of the electrophoresis are shown in Fig. 2.

**Figure 3:**
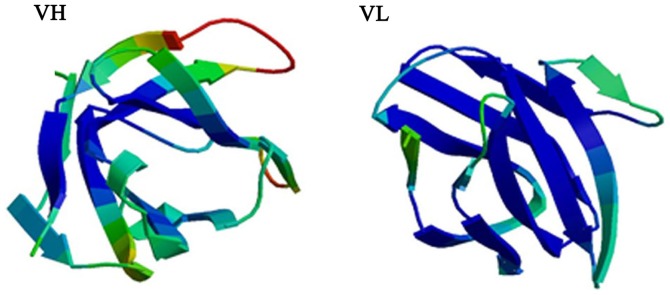
Single original antibody Swiss-models when linker n=0 and n=3. VH, heavy chain; VL, light chain.

**Figure 4:**
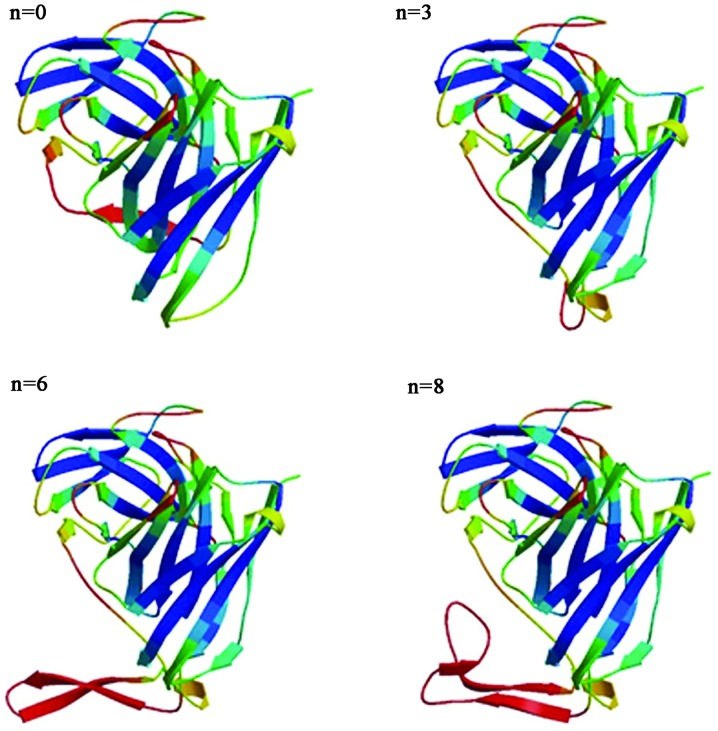
Single antibody models with linker lengths of n=6 and n=8.

The results of the VH and VL modeling in ScFv using the homologous modeling method are shown in Figs. 3 and 4. Generally, the length of the linker peptide of ScFv was between 10 and 40 amino acids long. The longer the length of the linker peptide, the less stable the structure of the ScFv. During the process of modeling, when n=9 (linker peptide length of 45 amino acids), modeling failed due to the low homology of the searched templates. Therefore, three-dimensional models were obtained when the n-value was between 0 and 8, and the results of the modeling are shown in Fig. 4. Each model had corresponding data for the VHn and VLn structure and data for the overall ScFv-n structure.

From the figures described above, there was a clear difference in the structural models between VH/VL alone and VH/VL when the linker was added, and the structure of the linker changed markedly as its length changed. Subsequently, the protein similarity algorithm, based on delamination of the spherical coordinates, was used to provide a contrast in the VH and VL similarity prior to and following addition of the linker. The results of the algorithm are shown in Table I.

Following several trials, the similarity data was relatively accurate and stable when the protein was divided into three layers. To guarantee the accuracy of the results, the protein was divided into 2, 3 and 4 layers. Subsequently, the mean similarity value of the data in each group was calculated and treated as the final result. As shown in Table I, when n=3 and n=6, the similarity data obtained was close to the original data of VH and VL. The similarity has indicated the effect of the linker peptide's length on the variable region structure to the ScFv, and the larger the value of the similarity, the lower the effect, which represented that the effect on the biological activity of a single-chain may be smaller.

Following the addition of the linker, the stability of VH and VL was analyzed again, based on the fore and aft distance and the diffusion radius. The results are shown in Table II.

**Figure 5:**
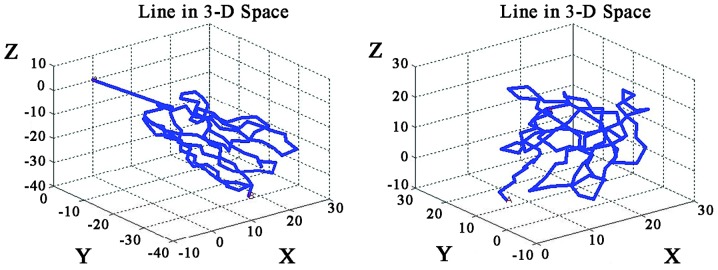
Original path of α carbon atom of a heavy chain and light chain.

**Figure 6:**
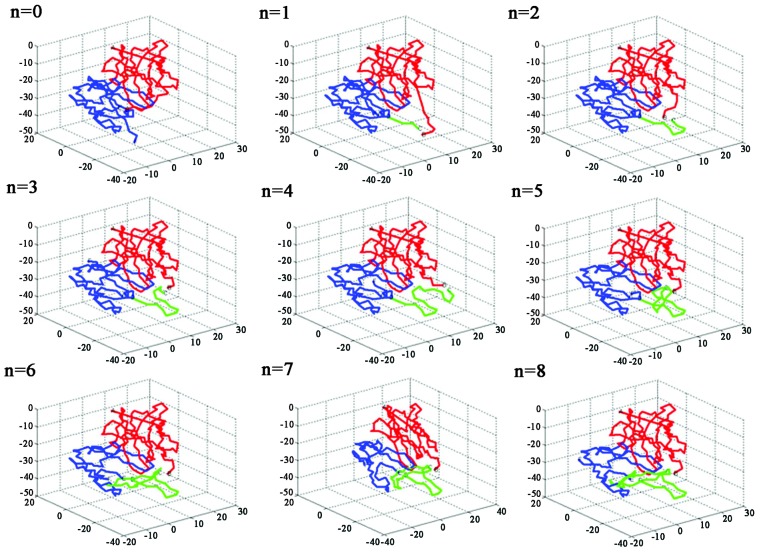
Path of α carbon atoms in a single-chain variable fragment when n= 1, 2, 3, 4, 5, 6, 7 and 8. Red indicates the heavy chain, blue indicates the light chain and green indicates the linker.

As shown in Table II, when n=6, the fore and aft distance (40.3791) of the VH was the closest to that of VH (39.9601); when n=3, in which the diffusion radius (25.6413) was also closest to the original data (25.2179). By contrast, in the VL, the fore and aft distance following addition of the linker reduced to almost half that of the original data, however, the diffusion radius increased. In addition, when n≤3, the fore and aft distance of the linker increased as the length of the peptide increased, whereas, when n>3, the fore and aft distance decreased due to folding of the lengthened peptide chain. The changes in the fore and aft distance also affected VH and VL. The position comparison in the space coordinates system of the original VH, VL and the α carbon atoms of the ScFv-n structure was established by MATLAB 2012a, as is shown in Figs. 5 and 6, which suggested the fore and aft distance change of VH and VL was as expected. This indicated that after adding linker peptides of different lengths, the change in the whole molecular radius was small.

## Discussion

From the comparative investigation performed in the present study, it was concluded that, in the spatial distribution of analogous proteins, the three-dimensional structure of proteins was more conserved than the primary sequence. The prediction of target protein spatial conformation is more reliable when performed in analogous proteins (10), and there have been several previous successes in predicting the structure of antibodies (10,11). Successful modeling of the 6B4 antithrombotic antibody by Fontayne *et al* (11) laid a foundation for the investigation of its antithrombotic activity and antigenicity Modeling of the three-dimensional structure of a protein can assist in the modification of the original antibodies on the basis of understanding its spatial structure and physiological functions, and it is significant for antibody engineering. ScFv molecule is a small molecule with weak immunogenicity and a rapid response time. It has no cumulative action in the kidney and its sharpness and clarity in tumor imaging is high, which enable it to be used as a vector and combined with medicine, isotopes and toxins (12). Single-chain antibody constructs belong to minimolecular antibodies. They are constructed by VH, VL and linker peptides, and are the smallest functional fragments of antibodies in retention of the antigen-binding site, which has significant theoretical and applicable value in the diagnosis and treatment of cancer.

In the present study, a series of methods, including modeling and bioinformatics, were used to examine and compare the structural changes in ScFv when the length of the linker peptide was altered. (Gly4Ser)n, as a linker peptide, is often selected in single-chain antibodies, among which 'Gly' represents glycine, 'Ser' represents serine and 'n' indicates the number of (Gly4Ser). The results of the present study revealed that, following addition of the linker, the similarity data for VH and VL was closest to that of the original data when n=3 and n=6, which indicated that ScFv-3 and ScFv-6 were closest to the original structure, as was their biological activity. In terms of stability, the fore and aft distance, and the diffusion radius of the VH and VL altered to different degrees following addition of the linker. Notably, when n=6, the fore and aft distance of the VH was closest to the original data, while that of the VL decreased by half. Compared with the original radius, that of VH and VL increased. When n=3, however, the diffusion radius of the VH was the closest to the original data. The majority of the results indicated the the significance of the effect of linkers n=3 and n=6 on VH and VL.

Despite the construction of ScFv or a single-strand bispecific antibody, the linker peptide cannot affect the folding of the structure of an antibody or its biological activities (13). Several studies have investigated the effect of the length of linkers on the activities of ScFv (14). Kikuchi *et al* (15) constructed an anti-CD47 bivalent single-chain antibody, MABLsc(Fv), by way of covalence, using linkers of 15 amino acids (Gly4Ser); Yan *et al* (16) found that a linker length of 15 amino acids was more favorable for the folding of antibodies, guaranteeing the affinity of the bivalent single-chain antibodies. Goel *et al* (17) successfully constructed bivalent single-chain antibodies with immune activity using a longer linker of 25 amino acids. The present study confirmed that when n=3 (linker with 25 amino acids), the two variable regions in the ScFv were closest to the original structure. The results also demonstrated that, when n=6, the effect of the linker on the structure of ScFv was smaller. Considering that the present study used a bioinformatics approach, and that there may be a certain level of error in molecular modeling, the results of the present study require confirmation using biological experiments. Whether a linker length of 30 amino acids is suitable for the favorable expression of biological activity in a single-chain antibody also requires further investigation.

The effects of structural changes of linker peptides in single-chain antibodies on the whole antibody molecules were examined using mathematical modeling and bioinformatics methods, providing a basis for further investigation of the preparation of single-chain antibodies.

## Figures and Tables

**Table I:** Results of similarity using a space spherical shell hierarchical matching algorithm.

Chain	Similarity			Average
	Layer 2	Layer 3	Layer 4	
VH0	0.8910	0.7653	0.6183	0.7582
VH1	0.9033	0.6320	0.7680	0.7678
VH2	0.9029	0.7931	0.5616	0.7525
VH3	0.9076	0.8376	0.5866	**0.7773**
VH4	0.9073	0.8198	0.5866	0.7712
VH5	0.9083	0.8299	0.5825	0.7736
VH6	0.9085	0.8299	0.5825	**0.7736**
VH7	0.9085	0.8258	0.5861	0.7735
VH8	0.9085	0.8258	0.5861	0.7735
VL0	0.9482	0.9723	0.8021	0.9075
VL1	0.9515	0.9726	0.7992	0.9078
VL2	0.9515	0.9729	0.7992	0.9079
VL3	0.9515	0.9734	0.7997	**0.9082**
VL4	0.9515	0.9726	0.7992	0.9078
VL5	0.9515	0.9729	0.7992	0.9079
VL6	0.9515	0.9734	0.8018	**0.9089**
VL7	0.9507	0.9734	0.7997	0.9079
VL8	0.9507	0.9734	0.7997	0.9079

Numbers in bold indicate a high degree of similarity. VH, heavy chain; VL, light chain.

**Table II:** Changes of the fore and aft distance and radius of VH, VL and linker prior to and following addition of the linker.

Chain	Fore and aft distance	Radius	Length of linker	Fore and aft distance of linker
VH0	32.9989	23.2127		
VH1	42.3431	35.2561		
VH2	37.1628	26.1484		
VH3	41.6467	25.6413		
VH4	38.8902	27.3090		
VH5	40.5697	28.6009		
VH6	40.3791	28.3365		
VH7	40.8331	28.1269		
VH8	40.8454	28.1225		
VH	39.9601	25.2179		
VL0	22.3826	25.8404	0	–
VL1	19.0852	25.8461	5	14.021
VL2	19.0833	25.8452	10	13.5976
VL3	19.0739	25.8434	15	17.3828
VL4	19.0852	25.846	20	25.2548
VL5	19.0740	25.8428	25	16.7704
VL6	19.0659	25.8402	30	16.5636
VL7	19.0846	25.8444	35	17.7174
VL8	19.0745	25.8442	40	18.0869
VL	36.7271	22.6299	–	–

VH, heavy chain; VL, light chain.
